# RETRACTION: Molecular Mechanism Whereby Paraoxonase‐2 Regulates Coagulation Activation Through Endothelial Tissue Factor in Rat Haemorrhagic Shock Model

**DOI:** 10.1111/iwj.70641

**Published:** 2025-04-23

## Abstract

**RETRACTION:** XuJ.‐H. , LuS.‐J. , WuP. , KongL.‐C. , NingC. , and LiH.Y. , “Molecular Mechanism Whereby Paraoxonase‐2 Regulates Coagulation Activation Through Endothelial Tissue Factor in Rat Haemorrhagic Shock Model,” International Wound Journal 17, no. 3 (2020): 735–741, 10.1111/iwj.13329.32090497 PMC7949017

The above article, published online on 23 February 2020, in Wiley Online Library (http://onlinelibrary.wiley.com/), has been retracted by agreement between the journal Editor in Chief, Professor Keith Harding; and John Wiley & Sons Ltd. An Expression of Concern had previously been published to alert readers to concerns regarding the correct methodology to measure PON2 [https://doi.org/10.1111/iwj.13614]. Following an investigation by the publisher, all parties have concluded that this article was accepted solely on the basis of a compromised peer review process. The editors have therefore decided to retract the article. The authors did not respond to our notice regarding the retraction.

**RETRACTION:** J.‐H. Xu , S.‐J. Lu , P. Wu , L.‐C. Kong , C. Ning , and H.Y. Li , “Molecular Mechanism Whereby Paraoxonase‐2 Regulates Coagulation Activation Through Endothelial Tissue Factor in Rat Haemorrhagic Shock Model,” International Wound Journal 17, no. 3 (2020): 735–741, 10.1111/iwj.13329.32090497 PMC7949017

The above article, published online on 23 February 2020, in Wiley Online Library (http://onlinelibrary.wiley.com/), has been retracted by agreement between the journal Editor in Chief, Professor Keith Harding; and John Wiley & Sons Ltd. An Expression of Concern had previously been published to alert readers to concerns regarding the correct methodology to measure PON2 [https://doi.org/10.1111/iwj.13614]. Following an investigation by the publisher, all parties have concluded that this article was accepted solely on the basis of a compromised peer review process. The editors have therefore decided to retract the article. The authors did not respond to our notice regarding the retraction.

